# Dosimetric Deviations of Bragg-Peak Position Shifts in Uniform Magnetic Fields for Magnetic Resonance Imaging-Guiding Proton Radiotherapy: A Monte Carlo Study

**DOI:** 10.3389/fpubh.2021.641915

**Published:** 2021-08-03

**Authors:** Xiaowa Wang, Hailun Pan, Qinqin Cheng, Xufei Wang, Wenzhen Xu

**Affiliations:** ^1^Department of Nulcear Science and Technology, Institute of Modern Physics, Fudan University, Shanghai, China; ^2^Key Laboratory of Nuclear Physics and Ion-Beam Application (MOE), Fudan University, Shanghai, China; ^3^Shanghai Proton and Heavy Ion Center, Shanghai, China; ^4^Shanghai Engineering Research Center of Proton and Heavy Ion Radiation Therapy, Shanghai, China; ^5^Shanghai Advanced Research Institute, Chinese Academy of Sciences, Shanghai, China

**Keywords:** proton pencil beam, simulation mode, proton dose changes, magnetic fields intensity, FLUKA simulation

## Abstract

**Objective:** To investigate dosimetric deviations in scanning protons for Bragg-peak position shifts, which were caused by proton spiral tracks in an ideal uniform field of magnetic resonance (MRI) imaging-guided proton radiotherapy (MRI-IGPRT).

**Methods:** The FLUKA Monte-Carlo (MC) code was used to simulate the spiral tracks of protons penetrating water with initial energies of 70–270 MeV under the influence of field strength of 0.0–3.0 Tesla in commercial MRI systems. Two indexes, lateral shift (marked as *WD*) perpendicular to the field and a penetration-depth shift (marked as Δ*DD*) along the beam path, were employed for the Bragg-peak position of spiral proton track analysis. A comparison was performed between MC and classical analytical model to check the simulation results. The shape of the 2D/3D dose distribution of proton spots at the depth of Bragg-Peak was also investigated. The ratio of Gaussian-fit value between longitudinal and transverse major axes was used to indicate the asymmetric index. The skewness of asymmetry was evaluated at various dose levels by the radius ratio of circumscribed and inscribed circles by fitting a semi-ellipse circle of 2D distribution.

**Results:** The maximum of *WD* deflection is 2.82 cm while the maximum of shortening Δ*DD* is 0.44 cm for proton at 270 MeV/u under a magnetic field of 3.0 Tesla. The trend of *WD* and Δ*DD* from MC simulation was consistent with the analytical model, which means the reverse equation of the analytical model can be applied to determine the proper field strength of the magnet and the initial energy of the proton for the planned dose. The asymmetry of 2D/3D dose distribution under the influence of a magnetic field was increased with higher energy, and the skewness of asymmetry for one proton energy at various dose levels was also increased with a larger radius, i.e., a lower dose level.

**Conclusions:** The trend of the spiral proton track under a uniform magnetic field was obtained in this study using either MC simulation or the analytical model, which can provide an optimized and planned dose of the proton beam in the clinical application of MRI-IGPRT.

## Introduction

Proton radiotherapy, an advanced form of cancer treatment, can fulfill the aim of radiotherapy to achieve better dose conformality than photons due to the Bragg peak. It results in irradiating the target uniformly with the prescription dose, while sparing the adjacent healthy tissues and critical organs (1) nearby to the target. The treatment modality of proton radiotherapy reduces the integral dose over the body of patients due to a finite penetration of protons. However, proton radiotherapy is more susceptible to uncertainties of the geometrical variations, such as a misalignment of setup, the motion of the target, and also of inter-fractional anatomical changes, due to the limited knowledge of patient anatomy during the initial CT simulation during treatment planning (2, 3). Imaging-guided proton radiotherapy (IGPRT) is implemented to reduce susceptible uncertainties to compensate for any unexpected target changes, thus sparing the healthy tissues (4) with proper safety margins in the treatment planning protocols. Magnetic resonance imaging (*MRI*) systems provide good contrast of soft tissues between the tumor and organs at risk (OARs) (5–9); it is a prime candidate for IGPRT with several advantages over other imaging modalities. Compared to the computed tomography (*CT*) imaging, several studies demonstrated the advantages of MRI-based imaging-guided proton radiotherapy (IGRT) (5–13). The MRI-IGPRT using protons can reduce uncertainties in the proton therapy process that result in lower doses to the normal tissues.

However, the spiral track of the proton path from the Lorentz force in a magnetic field of MRI made a dose deviation on the delivered 2D/3D distribution by shifting the Bragg-peak position (14–19), which depends on the gantry angle leading to different incident direction and strength of the magnetic field in MRI. In this study, the characteristics of a tracked spiral proton path and induced 2D/3D dosimetry deviation under an ideal uniform magnetic field were investigated using the FLUKA Monte Carlo (MC) simulation with a built-in magnetic field module (20). The incident energy range was 70–270 MeV/u and perpendicular to the magnetic field of strength of 0.0–3.0 Tesla. The previous study employed an analytical model to evaluate the Bragg-peak position shift in the spiral tracks and performed a comparison with the MC simulation (21). The magnitude of asymmetry of maximum planar dose distributions was also investigated for each energy under the field strength of 3.0 Tesla under different dose levels.

This study was implemented for the clinical application of MRI-IGPRT. Accurate 2D/3D dose distribution of employed energies over different field strengths was generated first, and then the shift of Bragg-peak position in terms of the lateral shift (*WD*) perpendicular to the field and the penetration-depth shift (Δ*DD*) along the beam path was evaluated. The values of *WD* and Δ*DD* from MC simulation were also compared with that from an analytical model for proton energy over different field strengths. The asymmetric spot shape was investigated by the ratio of Gaussian-fit values between longitudinal and transverse major axes at the planar maximum dose distribution of each proton energy over 3.0 Tesla field strengths. Fitting a semi-ellipse circle of 2D distribution at each dose level, the skewness of asymmetry over various dose levels was also evaluated by the radius ratios of circumscribed and inscribed circles.

## Materials and Methods

### Simulations Used FLUKA Monte-Carlo Code With Shifts of Bragg-Peak Positions

**Figure 1:**
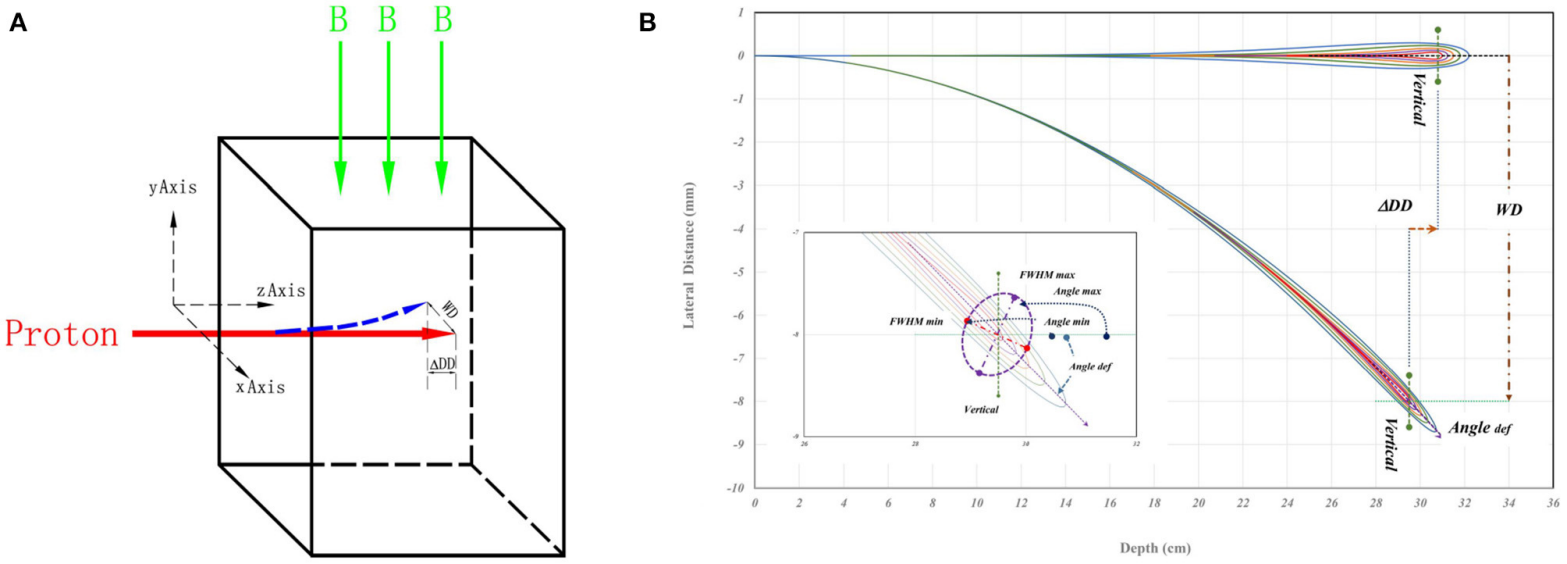
**(A)** A schematic drawing of simulation geometry with indicated directions of beam entrance and magnetic field. The proton beam vertically enters the water phantom from left to right, and the red arrow represents the beam. The green arrow is the direction of the magnetic field. **(B)** A schematic drawing for proton dose distributions as a straight path without magnetic field, and as a spiral track under the influence of magnetic field in a cut plane over the directions of beam entrance and the magnetic field. The *WD* and Δ*DD* were the shift of Bragg-peak position. The “*Angle def* ” is the deflection angle of spiral track beam central axis the. In the insert of **(B)**, the maximum and minimum of the full width at half maximum (FWHM) and the major and minor axes of elliptical 2D distribution at the Bragg-peak location. The Angle max and Angle min are the angles of maximum and minimum full-width-half-maximum in the major and minor axes of doses passing the location of Bragg-peak. Details are in the text.

FLUKA (2011.2x) was implemented to simulate the interaction between active pencil-beam-scanning (PBS) proton and the magnetic field (20). Each simulated PBS proton beam entered a water phantom perpendicular to the uniform magnetic field as shown in Figure 1A. The red arrow indicates the entering PBS proton, while the green arrow shows the direction of the magnetic field. In Figure 1B, a schematic drawing for proton dose distribution, the straight path means without magnetic field and the spiral track means under the influence of a magnetic field in a cut plane over the directions of beam entrance and the magnetic field. The *WD* and Δ*DD* were used to evaluate the shift of Bragg-peak position in direction of transverse (i.e., perpendicular) and longitudinal (i.e., along) beam paths for the shifts. The “*Angle def* ” was the deflection of spiral track beam central axis. The insert of Figure 1B presents the characteristics for the 3D dose distributions of spiral proton tracks. Details of these parameters will be described later.

In MC simulations, protons were transported in a water phantom with a size of 20 × 20 × 35 cm^3^ (x, y, and z directions of the phantom geometry, and the distance in z direction is larger than the range of 270 MeV/u incident energy). Parameters of the hadron therapy to a 100 keV limit and cutoff range of transport 0.001458 mm were used in simulation for sufficient accuracy. The water phantom was binned to obtain 0.1 × 0.02 × 0.02 (z, x, and y directions of the phantom geometry) mm^3^ voxels. 3D dose distribution was simulated with 1 × 10^10^ particle histories in each spot with 5 mm sigma of 2D Gaussian lateral distribution at the entrance of the water phantom. The directional divergence of proton space was neglected in simulations because it was minimal in comparison with the lateral scattering spreading in water by the multiple Coulomb scattering (22). The statistical error of each simulation was <2% for local maximum value at the depth before the Bragg-peak. The statistical error of the results within 3 mm before and after the Bragg peak was controlled within 0.2%, while that of the low dose beyond 3 mm of Bragg peak was controlled within 4.0%. Simulations were performed with the proton energy of 70, 80, 90, 100, 110, 120, 130, 140, 150, 160, 170, 200, and 270 MeV/u under the magnetic field strength of 0, 0.5, 1.0, 1.5, 2.0, 2.5, and 3 Tesla, which covered the typical parameters used in proton radiotherapy. *WD* and Δ*DD* of spiral proton tracks were analyzed in each MC simulation.

### The Mathematical Formula of an Analytical Model to Calculate *WD* and Δ*DD*

Following the reference (21), the shift (*WD* or Δ*DD*) of Bragg-peak position can be calculated by the mathematical formula as shown in Equations (1)–(3). For a proton with kinetic energy *E*_0_, charge *q* = *1*, and rest mass *m* entering perpendicularly to a magnetic field strength *B*, the shift of *WD*can be calculated by either Equation (1) or (2), which correspond to non-relativistic and relativistic cases, respectively. For protons with energy of 70, 80, and 90 MeV/u, only Equation (1) was used to calculate the *WD* by considering the energy effect, while both Equations (1) and (2) were used to calculate the *WD* in an interval of 10 MeV for protons with energy 100–170 MeV.

(1) $$\begin{array}{r} WD=\frac{7}{12}\frac{qB\alpha^{2}}{\sqrt{2m}}E_{0}^{3} \end{array}$$

(2) $$\begin{array}{r} WD=\frac{7}{12}\frac{qB\alpha^{2}}{\sqrt{2m}}E_{0}^{3}\left(1-\frac{3}{8}\left(\frac{E_{0}}{2mc^{2}}\right)\right) \end{array}$$

Where *p* ≈ 1.75 and α ≈ 2.43×10^−3^ MeV^−*p*^ cm in water for the fit parameter of proton stopping power in water.

The shift of Δ*DD* can be calculated by Equation (3).

(3) $$\begin{array}{r} \Delta DD=\frac{q^{2}B^{2}\alpha^{3}E_{0}^{3p-1}}{2m}\left(\frac{2p^{2}}{\left(4p-1\right)\left(3p-1\right)}\right) \end{array}$$

The *WD* and Δ*DD* calculated with the upper equations at certain conditions were compared with corresponding values of MC simulations, and the reverse equations can be used to determine the required energy according to *WD* and Δ*DD*.

### The Asymmetries of 2D/3D Dose Distributions Induced by Magnetic Fields

In addition to the shifts of Bragg-peak position, the 2D spot-shape become asymmetric under the influence of a magnetic field, and the asymmetry especially can be visually seen for the planar dose at the Bragg-peak. To evaluate the skewness of asymmetry of simulated planar 2D distribution, an analysis process using MATLAB (23) platform was used to investigate the referred macroscopic and microscopic aspects in this study. The analysis process includes: (1) Performing a Gaussian fit for obtaining the sigma of 1D distribution in the direction of transverse (*x*) and longitudinal (*y*) axes at the plane of the planar dose distributions; (2) Analyzing the ratio of obtained sigma in *x/y* axes with a field strength of 3.0 Tesla as a function of energy; and (3) Investigating the differences in the area of encompassed ellipse under the magnetic field concerning a standard circle without magnetic field, while the size of each standard circle in the plane depends on the dose level.

The processes of 1 or 2 analyze the data only in the *x-* or *y-*axis itself and refer to the macroscopic aspect. Because the minimum/maximum axis of ellipse cannot be on *x/y* axes, each ellipse can be fitted by minimalizing the area between an inscribed circle (*IC*) and a circumscribed circle (*EC*) where the IC is the maximum radius with the ellipse and the EC the minimum radius outside the ellipse. The process of 3 refers to the macroscopic aspect by involving the asymmetry at a dose level.

Although the deflection of spiral tracks causing the shifts of Bragg-peak position and the 2D dosimetric deviations in macroscopic and microscopic aspects described above, the major and minor axes of elliptical 2D distribution at the Bragg-peak location may not align with *x/y* axes related to the beam direction as shown in the insert of Figure 1B. The MATLAB code was used to analyze the FWHM of each line passing through the maximum dose point of the Bragg peak in each simulated 3D distribution. It is that the spatial positions of lines having maximum FWHMmax and minimum FWHMmim of each simulated 3D dose distribution were located. Based on the orientations of FWHM lines, the rotation angles of FWHM_max_ and FWHM_mim_ lines to the beam incidence are the Angle_max_ and Angle_min_ as indicated in the insert of Figure 1B.

## Results

### The Shifts of Bragg-Peak Positions

#### The MC Simulations and the Analytical Model

**Figure 2:**
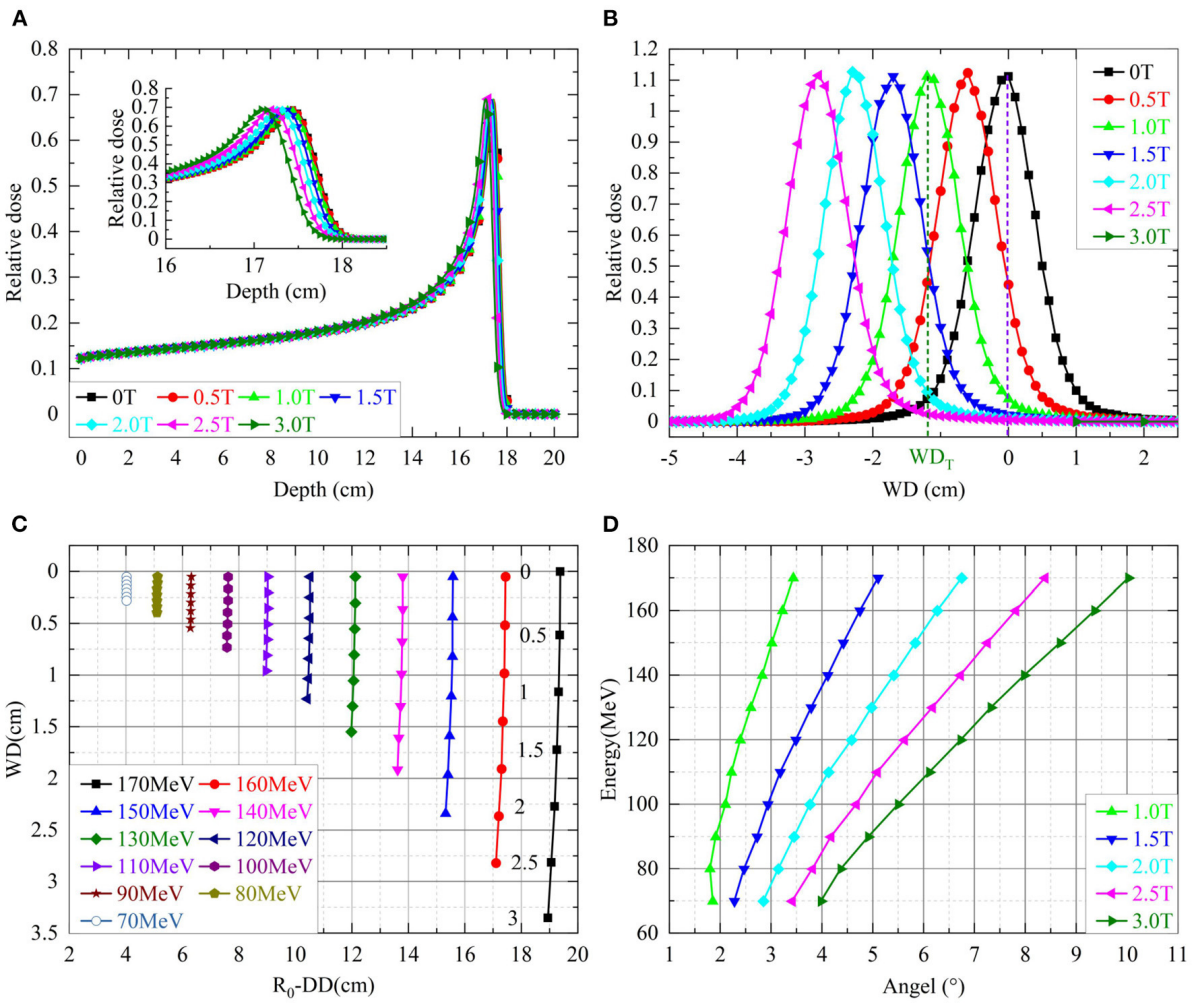
**(A)** Shows the simulated depth doses of protons with 170 MeV over different field strengths. The insert shows the details of depth doses near the Bragg-peak. **(B)** Shows the shifts of *WD* for various energies with different field strengths. The numbers next to the shifts of 170 MeV indicated corresponding field strengths. **(C)** Shows the lateral dose profiles of protons with 170 MeV over different field strengths in the axis of *WD*. **(D)** Shows the beam deflection angles for incidence beam direction.

Each simulated depth dose was normalized to the maximum dose at the phantom surface. Figure 2A shows simulated depth doses for protons with an energy of 170 MeV/u under different field strengths. The insert of Figure 2A shows the shifts of Δ*DD*. Figure 2B shows the shifts of *WD* for various energies with different field strengths. Figure 2C shows the lateral profiles shifting under various field strengths. Figure 2D shows the beam deflection angles, calculated from *WD* and Δ*DD*, concerning the incident beam direction.

Table 1 lists the percentage differences on *WD* from Equations of (1) or (2) to the simulations over proton energies of 100–170 MeV/u. Both smaller proton energy and a weaker magnetic field would result in a large percentage difference. The mean of percentage differences over listed energies for the Equations of (1) and (2) were 4.7 and 5.6%, respectively. However, the maximum absolute difference of *WD* between the analytical model and the MC simulation is around 0.5 mm.

**Table 1:** The percentage differences of *WD*for formulas 1 and 2 for simulations.

**Energy (*MeV*)**	**Formula 1 (%)**	**Formula 2 (%)**
100	9.81	11.61
110	6.88	8.93
120	4.92	6.95
130	3.76	5.35
140	3.11	4.08
150	2.93	3.18
160	2.90	2.52
170	3.23	2.10

Table 2 shows the difference between the analytical model of Equation (3) and the MC simulation for various proton energies under different field strengths. The maximum difference is 0.1 mm.

**Table 2:** Differences ofΔ*DD* between the analytical model of the Equation (3) and the MC simulation.

**Energy (MeV)**	**B = 1.5T**		**B = 2.0T**		**B = 2.5T**		**B = 3.0T**	
	**Simulation**	**Formula 3**	**Simulation**	**Formula 3**	**Simulation**	**Formula 3**	**Simulation**	**Formula 3**
	**(mm)**	**(mm)**	**(mm)**	**(mm)**	**(mm)**	**(mm)**	**(mm)**	**(mm)**
100	0.2	0.2	0.2	0.2	0.4	0.4	0.4	0.5
110	0.2	0.2	0.2	0.2	0.4	0.4	0.6	0.6
120	0.2	0.2	0.4	0.4	0.6	0.6	1	1
130	0.4	0.3	0.6	0.6	1	1	1.4	1.4
140	0.4	0.3	0.8	0.8	1.4	1.3	1.8	1.8
150	0.6	0.6	1.2	1.1	1.8	1.8	2.6	2.7
160	1	0.9	1.4	1.5	2.4	2.4	3.4	3.3
170	1.2	1.1	2	1.9	3.2	3.1	4.4	4.4

#### Applications of Analytical Model for Desired Δ*DD* and *WD* in the MRI-IGPRT

Based on the position relationship between an OAR and the target, required Δ*DD* and *WD* can be determined. The Equations of (4) and (5) derived from the revision of Equations (1) and (3) can be used to determine the proton incident energy with a specific field strength to achieve an optimized delivered doses for MRI-IGPRT.

(4) $$\begin{array}{r} E_{0}={\left(WD\times \frac{12}{7}\frac{\alpha^{-2}\sqrt{2m}}{qB}\right)}^{\frac{1}{3}} \end{array}$$

(5) $$\begin{array}{r} E_{0}={\left(\Delta DD\times \frac{\alpha^{-3}•2m}{q^{2}B^{2}}\frac{\left(4p-1\right)\left(3p-1\right)}{2p^{2}}\right)}^{\frac{1}{3p-1}} \end{array}$$

For example, to meet the required position of *OARs* for avoidance required a *WD* = 2.0 cm or Δ*DD* = 0.25 *cm* under a 3.0 Tesla of field strength; calculated incident energy E_0_ of the protons by formula 4 or 5 are 141 MeV/u or 148 MeV/u, respectively. The energy of protons was input to the MC simulation with a 3.0 Tesla of field strength. The MC simulated *WD* and Δ*DD* outcomes were 2 cm and 0.24 cm, respectively. Similar results validated that Equations (4) and (5) can be used for calculating the incident proton energy of required *WD* and Δ*DD*. In clinical practice, this approach by proper proton energy under a specified field strength could achieve the desired delivered doses of MRI-IGPRT.

### The Asymmetries of 2D/3D Dose Distributions Induced by Magnetic Fields

#### Characteristics of Asymmetric Planar 2D Dose Distributions

**Figure 3:**
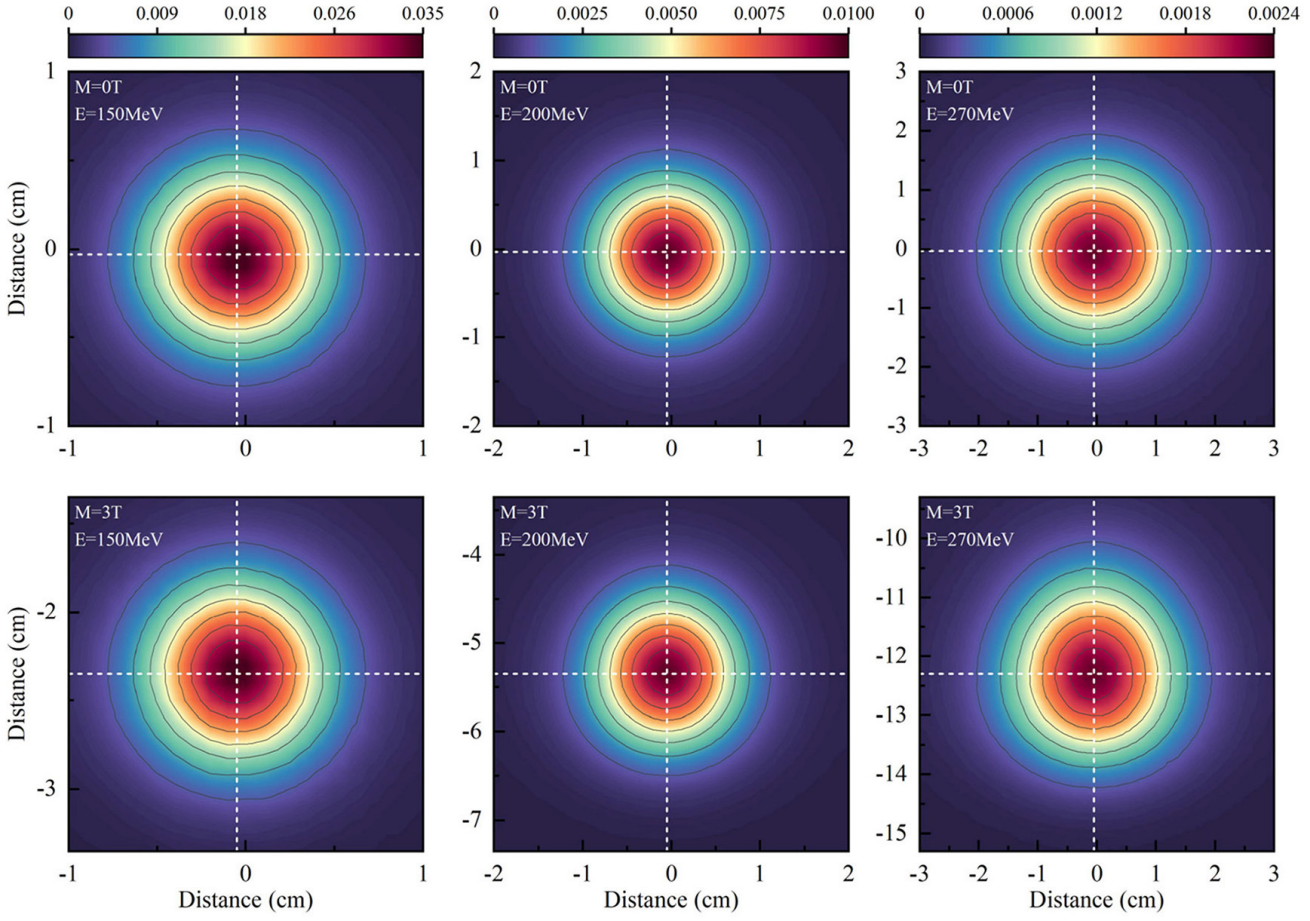
Shows the planar dose distributions at the Bragg-peak positions for protons without magnetic fields at the panels and under a field strength of 3.0 Tesla.

Symmetric planar dose distributions at the depth of Bragg-peak without a magnetic field were observed in the top panels of Figure 3. However, asymmetric planar distribution for protons of 270 MeV/u appear under a field strength of 3.0 Tesla.

**Figure 4:**
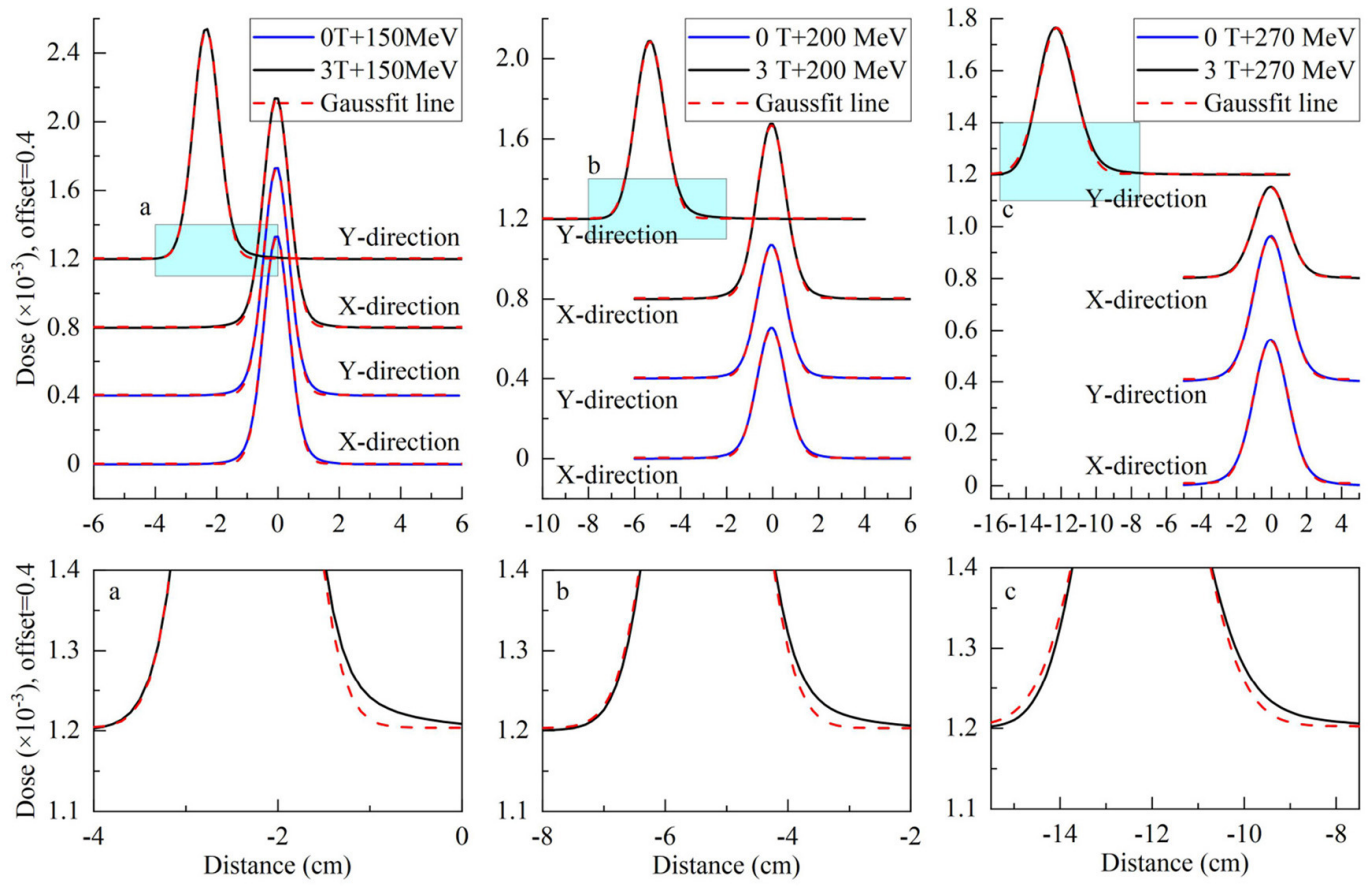
Top panels show extracted *x-*transverse and *y-*longitudinal 1D distributions in the solid blue curve with Gaussian fit curves in the dotted red curve for 2D dose distributions in Figure 3. Bottom panels show the detail of *y-*longitudinal distributions at lower dose levels.

To analyze the characteristics of asymmetric planar distribution, the 1D distribution at the *x-*transverse and *y-* longitudinal axes from each panel of Figure 3 were extracted and presented in Figure 4. Each extracted 1D profile of the *x-* or *y-*axis was fit by Gaussian function. In the absence of a magnetic field, each 1D profile for different energies was symmetrical in comparison to each fit curve. The *x-*transverse profiles for various energies were still symmetrical even under a field strength of 3.0 tesla. However, *y-*longitudinal profiles were visually asymmetric at lower dose levels. These results indicated the unidirectional distortion of proton dose distributions under the magnetic fields.

**Figure 5:**
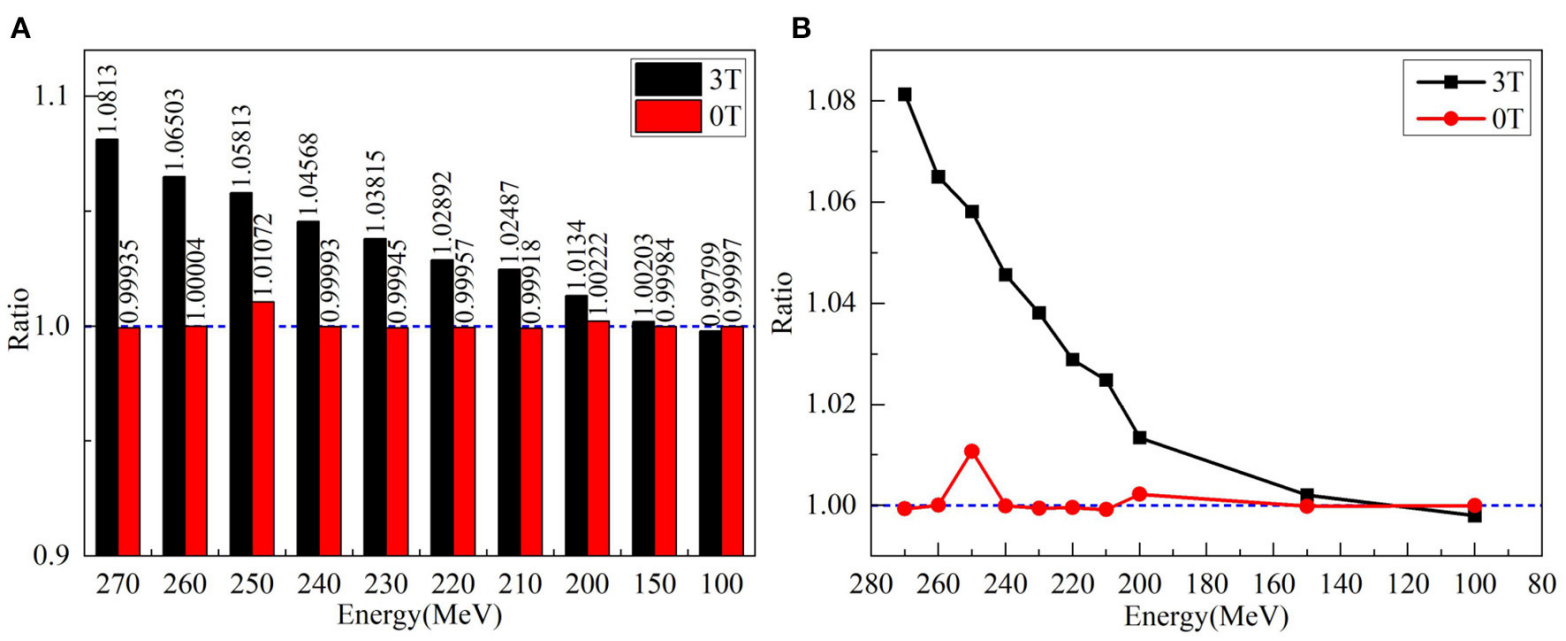
**(A)** Left panel shows the ratios of sigma widths on the Gaussian fits of *x-* and *y-*profiles for various proton energies without a magnetic field and under 3.0 Tesla. **(B)** The right panel showed the ratio of left panel by circles without magnetic field and by squares with a field strength of 3.0 Tesla.

Although the *x-*transverse profile is symmetric, the *y-*longitudinal profile is asymmetric under the magnetic fields. The changes of sigma widths are also different between *x-* and *y-*profiles under different field strengths. To present the ratio of sigma, widths on the Gaussian fits of *x-* and *y-*profiles were obtained and plotted in Figure 5 for various proton energies without magnetic field and under 3.0 Tesla. In the absence of a magnetic field, the axis ratios of longitudinal–transverse proton dose distributions were almost identical. Contrastively, in the presence of a magnetic field, the ratios increased with the enhancing energy, especially at energies > 150 MeV/u.

**Figure 6:**
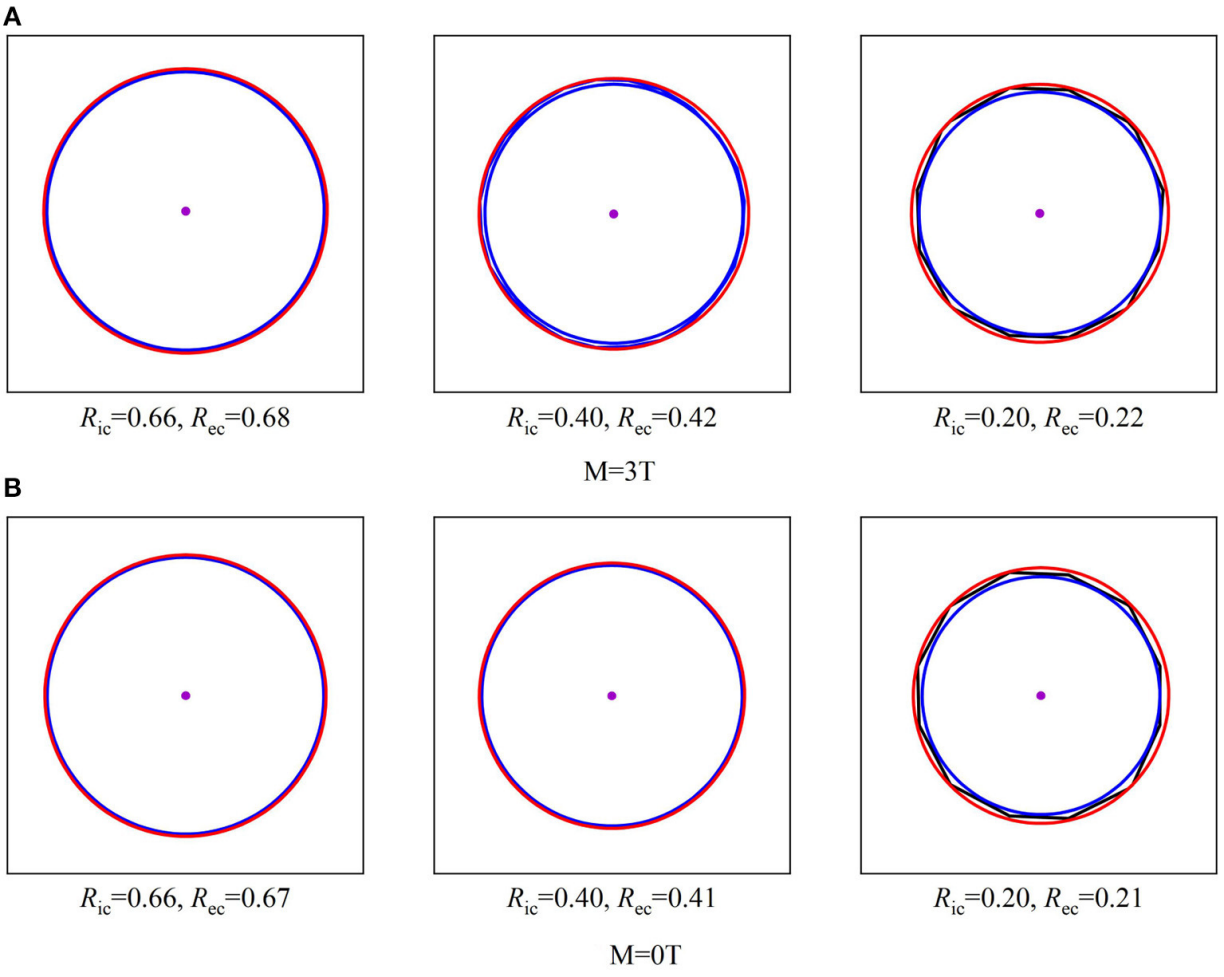
Shows three sizes of isodose curves for 150 MeV protons over three dose levels under a 3.0 Tesla field strength with **(A)** and without a magnetic field **(B)**. The center point of each plot indicates the maximum dose at Bragg-peak position of each simulation. Each isodose curve was fit for a minimized area between the inscribed *IC* and *EC* circumscribed circles as described in the text to obtain the radii of *IC* and *EC*. When E = 150MeV.

**Figure 7:**
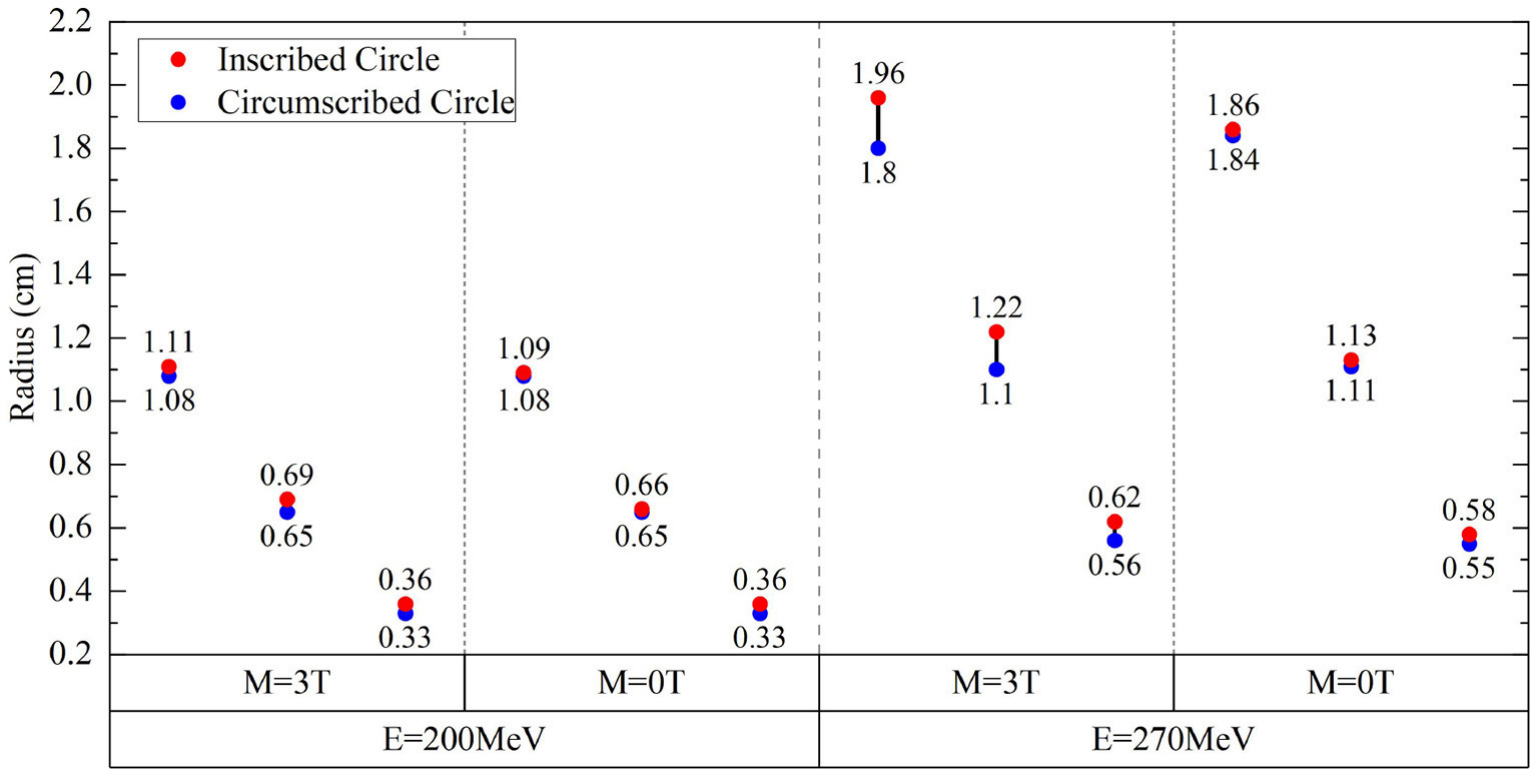
Radius differences of fitted *IC/EC* circle for isodose curves for protons of 200 and 270 MeV under the field strengths of 0.0 and 3.0 Tesla.

Besides the ratios of sigma widths varying with the proton energy under different field strengths, the changes of sigma widths are different for the isodose curves at different dose levels as shown by the different size of isodose curves in Figure 6. Because the change of sigma width of each isodose curve is different between *x* and *y* axes, it results in an elliptical shape for each isodose curve under the influence of the magnetic field. By applying the fit process described above, the radii of IC/EC circles were obtained for each isodose curve to be plotted in Figure 7. The area differences of these circles exhibited a certain regularity in the presence of the magnetic field. The average radius differences for a field strength of 3.0 Tesla were twice those without a magnetic field.

**Figure 8:**
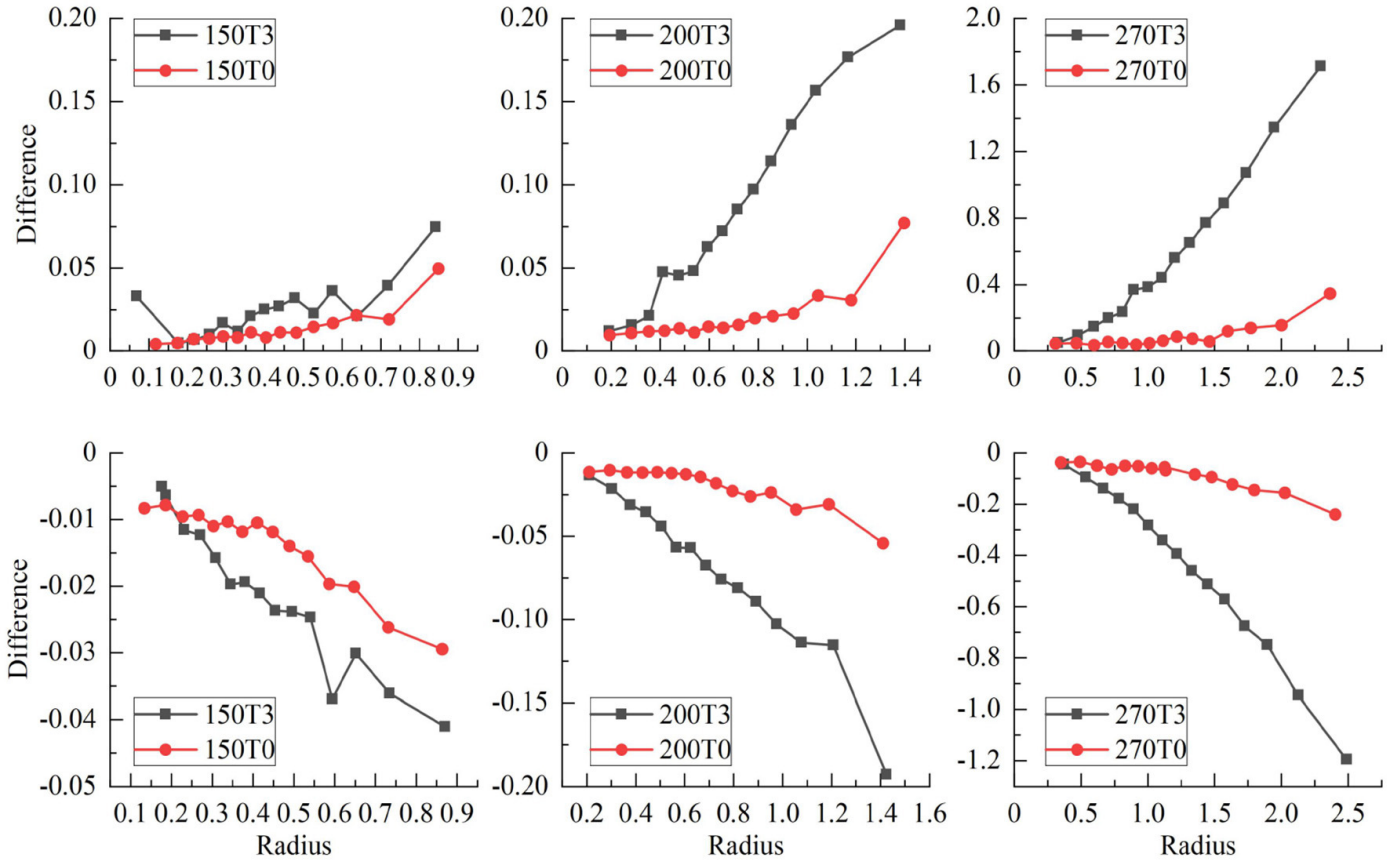
Shows the trend of radius difference between the IC and EC circles as a function of the radius in the standard circle for protons with energies of 150, 200, and 270 MeV.

Figure 7 shows the radii for fit IC/EC circles of three isodose curves for protons with energies of 200 MeV/u and 270 MeV/u under the field strengths of 0.0 and 3.0 Tesla. The difference between *IC* and *EC* radii increases as the size of the isodose curve increases, i.e., lower dose level. The effect of a magnetic field for 200 and 270 MeV/u protons was three and five times stronger than 150 MeV/u protons, respectively. Figure 8 shows the trend of radius difference between the *IC* and *EC* circles for protons with energies of 150, 200, and 270 MeV/u as a function of radius without as magnetic field. Many standard radii were at the lower dose level. A general trend for larger differences with increased standard radius was seen for all three energies.

#### The Deflection of Major Axes for Asymmetric 3D Dose Distributions

As shown in the insert of Figure 1B, the FWHM of each line passing through the maximum dose point of the Bragg peak were analyzed. The lines passing the maximum and minimum of FWHM were rotated away from its initial orientation; it was initially aligned with the vertical axis as indicated in Figure 1B. Due to the re-orientation of major and minor axes of 3D dose distribution for protons passing a magnetic field, the plane formed by the major and minor axes is a certain angle with the beam incident direction as the elliptical circle indicated in the insert of Figure 1B. Extracted rotation angles of Angle_max_ and Angle_min_ are listed in Table 3 for protons with energies of 150, 200, and 270 MeV under a field strength of 3.0 Tesla. The values of FWHM_max_ and FWHM_mim_ are also listed in Table 3 with the FWHM along the vertical axis. Notice that the maximum FWHM is not always along the vertical direction, but can be in a different direction.

**Table 3:** Dose calculations on different angle profiles.

**E (MeV)**	**FWHM_**max**_**	**Angle_**max**_**	**FWHM_**min**_**	**Angle_**min**_**	**FWHM_**vertical**_**
	**(cm)**	**(degree)**	**(cm)**	**(degree)**	**(cm)**
150	0.8212	1	0.6577	25	0.8122
200	1.3489	81	1.0512	31	1.3141
270	2.3167	83	1.7456	14	2.2304

## Discussion

The MRI-IGRPT can reduce the uncertainties in the radiotherapy process and improve the patient's positioning accuracy. However, the magnetic field significantly alternates the dose distributions of protons passing the magnetic field as shown in our results. In this study, details of the spiral proton track and the asymmetric 2D/3D dose distribution were investigated. The magnetic field not only induces the shift of Bragg-peak position as the spiral proton track, but also varies the 2D/3D dose distributions (11, 12, 14, 15). Induced asymmetric variation of 2D/3D dose distributions were evaluated on the ratio of Gaussian-fit values in the 2D dose distributions. The skewness of asymmetry at different dose levels by the differences between the circumscribed and inscribed circles was also studied. Finally, the rotation angles to the beam incidence for major axes were investigated for maximum and minimum FWHW in the 3D dose distribution.

To validate an analytical model for predicting simulated Δ*DD* and *WD* shifts of Bragg-peak position, the mathematic formula derived by Wolf and Bortfeld (21) was used in this study. The validation and verification were successfully conducted using the analytical model. With a validated analytical model, the mathematic formula by reversing the formula was derived. Based on the derived mathematic formula, the anatomical position of the target can be calculated based on the required proton incident energy, which can thus avoid the irradiation of OARs, aiming to achieve the purpose of optimizing proton radiotherapy.

Magnetic fields perturb more on the 2D/3D dose destitutions when the energy or/and magnetic field increases. Some existing research on magnetic fields and proton radiotherapy only described the change of proton dose or made preliminary dose calculations (14, 24, 25). Dosimetry deviation in the 2D/3D distribution were performed on the macroscopic to microscopic aspects with parameters such as the angles of defection and the orientation of major/minor axes of the plane. With the validated analytical model and details of characteristics of dosimetric deviations, the dose perturbation due to the magnetic field can be optimized to deliver desired doses to the treated target.

## Conclusion

The trend of the spiral proton track under a uniform magnetic field obtained in this study by using either MC simulation or the analytical model can provide optimized doses of the proton beam in the clinical application of MRI-IGPRT. Further developments of the analytical dose calculation algorithm are needed to model the asymmetric dose distribution of protons passing a realistic non-uniform magnetic field.

## Data Availability Statement

The original contributions presented in the study are included in the article/supplementary material, further inquiries can be directed to the corresponding author.

## Author Contributions

XuW and XiW proposed and designed the experiments. XiW and XuW carried out the experiments with the help of HP, QC, and WX. XiW drafted the manuscript and interpreted the data. XuW revised the manuscript. All authors contributed to the article and approved the submitted version.

## Conflict of Interest

The authors declare that the research was conducted in the absence of any commercial or financial relationships that could be construed as a potential conflict of interest.

## Publisher's Note

All claims expressed in this article are solely those of the authors and do not necessarily represent those of their affiliated organizations, or those of the publisher, the editors and the reviewers. Any product that may be evaluated in this article, or claim that may be made by its manufacturer, is not guaranteed or endorsed by the publisher.
